# The potential use of spectral electromyographic fatigue as a screening and outcome monitoring tool of sarcopenic back muscle alterations

**DOI:** 10.1186/1743-0003-11-106

**Published:** 2014-07-02

**Authors:** Thomas Kienbacher, Richard Habenicht, Christian Starek, Patrick Mair, Markus Wolf, Birgit Paul, Sara Riegler, Josef Kollmitzer, Gerold Ebenbichler

**Affiliations:** 1 Karl-Landsteiner-Institute for outpatient rehabilitation research, Porschestraße 29, Vienna A-1230, Austria; 2 Department of physical medicine and rehabilitation, Vienna Medical University, General Hospital of Vienna, Vienna, Austria; 3 TGM – School of Technology, Vienna, Austria; 4 University of Applied Sciences, Biomedical Engineering, Vienna, Austria; 5 Department of Psychology, Harvard University, Cambridge, MA, USA

**Keywords:** Back muscles, Fatigue, Electromyography, Sarcopenia, Power spectrum analysis

## Abstract

**Background:**

To examine whether or not median frequency surface electromyographic (MF-EMG) back muscle fatigue monitoring would be able to identify alterations in back muscle function in elderly muscles, if a protocol was used that allowed optimum standardization of the processes underlying electromyographic fatigue, and whether these tests were reliable from day to day.

**Methods:**

A total of 42 older (21 females; 67 (±10.5) years old) and 44 younger persons (19 females; 33 (±10) years) performed maximum isometric back extensions which were followed by one 30 s lasting 80% submaximum extension. Participants were seated on a dynamometer with their trunks 30° anteflexed, and they repeated all tests after 1-2 days and 6 weeks. SEMG was recorded bilaterally from the L1 (iliocostalis lumborum), L2 (longissimus), and L5 (multifidus) recording sites. Outcome variables included maximum back extension torque, initial MF-EMG (IMF-EMG), MF-EMG slope declines, and individual MF-EMG muscular imbalance scores. Two-factorial ANOVAs served to examine the age and gender-specific effects, and models from Generalizability Theory (G-Theory) were used for assessing retest-reliability.

**Results:**

Maximum back extension moment was non-significantly smaller in elders. IMF-EMG was overall higher in elders, with significant differences at the L5 recordings sites. In the elderly, MF-EMG fatigue declines were significantly smaller in L5, in the recording with the most negative slope, or if the slope of all electrodes was considered. Retest reliability was unanimous in young and older persons. ICC-type measurements from G-Theory of both the IMF and the fatigue slopes ranged from 0.7 to 0.85. Absolute SEM values were found clinically acceptable for the IMF-EMG, but relatively high for the fatigue slope declines.

**Conclusions:**

The MF-EMG fatigue method is able to elucidate alterations of aging back muscles. This method, thus, might be suggested as a potential biomarker to objectively identify persons at risk for sarcopenia. Considering the clinical relevance of the IMF-EMG relative to the MF-EMG slope declines, spectral EMG may also be used as an outcome monitoring tool in elderly populations.

## Background

Rapid growth of the aging population, the associated incidence of disability, and dramatically increasing health expenditures have all led to an increased scientific interest in the preventive and rehabilitative assessment of the neuromuscular functions in aging. It is well documented that advancing age is accompanied by a pronounced decrease in muscle mass and quality. Such structural and functional impairments to muscle relate to disability and morbidity and may create a considerable burden for many older persons [1,2]. Recent evidence suggests back muscle function to be of utmost importance in the preventive and rehabilitative assessment of balance and mobility performance in the elderly [3-5]. Thus, assessment tools that objectively, reliably and feasibly identify and assess sarcopenic back muscle function in elderly persons are highly warranted.

Static sustained back extension tests using a submaximum load defined by electromyographic criteria have successfully been used to quantify back muscle capability and differentiate normal from impaired back muscle function [6,7]. Surface electromyography (EMG) monitors information that relates to the presence of muscle fatigue even during limited submaximum contractions and has the advantage of assessing muscle capability independent of its maximum performance. Based on strong relationships between muscle fiber diameter and its conduction velocity [8] and between conduction velocity and the EMG spectral component [9,10], the spectral EMG variables like the median frequency (MF) and its related changes over time are likely a good indication of muscle composition and have been suggested to measure muscle composition and muscle weakness non-invasively [11]. The MF refers to the frequency value that separates the energy of the EMG power frequency spectrum into two equal halves.

The current understanding of how aging affects the properties of muscle endurance and the physiological phenomenon of local muscle fatigue remains incomplete. Recent findings from large extremity muscle groups of elders suggest that, unlike muscle strength, muscle endurance is relatively preserved [12]. This was further evidenced by a paradoxically higher EMG-related resistance to fatigue, if sustained or intermittent isometric contractions of extremity muscles at relative intensities and limit of fatigue were monitored [13,14]. Such findings are in agreement with observations of an accelerated loss of type II fibers [15,16] and motor unit remodeling with aging, in which type II fibers are re-innervated by collateral sprouting of axons innervating the slow motor units [17]. Thereby, re-innervated type II fibers likely become type I fibers [18].

Whether or not spectral EMG would also be sensitive enough to identify age-specific preservations of back extensor endurance in elderly persons has not been well investigated. The lumbar extensors have a relatively high proportion of slow twitch, fatigue resistant fibers [19] that are larger than those of extremity muscles. Larger muscle fibers dispose of higher muscle fiber conduction velocities and in turn lead to higher median frequencies (MF) [10]. Considering the sarcopenic changes within muscles that may occur with advancing age, it is not surprising that those two studies that compared isometric spectral EMG back muscle fatigue between young and elderly healthy subjects overall failed to demonstrate relevant age-specific differences [13,20]. However, one of these studies observed in a sub-analysis of the data clearly steeper MF-EMG fatigue slopes in young subjects, if the relative back extension load was as high as 70% of the maximum load [13].

The validity and sensitivity of the back extensor fatigue protocols, which intended to examine age-specific alterations with the spectral EMG method that has been used so far, may be doubted as the EMG underlying fatigue process has unlikely been standardized in a sufficient way. In fact, various sources, like the percentage of motor units recruited in a contraction, the type of back muscles tested, the task specificity of back muscle testing [6,21], and the variability related to back muscle blood perfusion during moderately to mildly severe loaded submaximum contractions [8], are all known to affect the spectral EMG and fatigue-related processes, thereby decreasing the validity of the spectral EMG fatigue measures [8,11].

Test-retest reliability of the spectral EMG fatigue method is essential to determine the most stable measures for repeated fatigue assessments and to demonstrate their potential clinical usefulness. Whereas retest reliability of back extensor spectral EMG fatigue has repeatedly been studied in young healthy persons with mixed results [22-25], no such data seem available for elderly persons. Reproducibility of spectral EMG fatigue measurements from elders could differ from those of younger adults due to altered (lower) initial MF (IMF) and fatigue slope decline values, as well as reduced consistency of measurements from day to day, since these persons may be more vulnerable to day to day fluctuations in physical and mental health.

The MF-EMG fatigue back extensor testing method has excellent potential to become an effective screening tool for sarcopenia. Using novel EMG techniques, these measures are easy to perform, time and cost efficient, and free of side effects. Therefore, the purpose of this study was to investigate whether 1) MF-EMG fatigue of the back extensor muscles, using a test protocol that allowed for an optimum standardization of the physiologic processes underlying electromyographic fatigue, would differ between persons older and younger than 50 years of age, 2) the test-retest reliability of these MF-EMG fatigue measures was adequate for clinical use in elders, and 3) whether these reliability data were comparable to those of young persons.

## Methods

### Participants

A total of 86 asymptomatic volunteers who were recruited through personal contacts of the examiners, advertising presentations in leisure time institutions for elderly, and companies in the area close to the Karl-Landsteiner institute of outpatient rehabilitation research as well as staff from an outpatient rehabilitation institute were enrolled into the study. Of these, 44 participants (19 women) were between 18 and 49 years old, and another 42 (21 women) were 50 – 90 years old.

Physicians specialized in physical & rehabilitation medicine screened all volunteers. All participants were healthy, exhibited normal physical activity (but did not participate in competitive sports more than 2 times per week), and were free of any risk factors that would preclude them from participating in exercise. Exclusion criteria were 1) inability to follow German speaking instructions, 2) more than 5 mild back or referring back pain episodes (VAS > 30) lasting more than 2 days each within the past year, 3) a history of spine surgery or any kind of specific disease, 4) pregnancy, 5) any medical condition that might interfere with maximum strength or submaximum endurance testing, or 6) a BMI exceeding 35 kg/m^2^.

The study protocol was acknowledged by the Ethics’ committee of the city of Vienna. Before inclusion, all participants received oral and written information about the study and signed an informed consent. The data collection was carried out in accordance with the directives given in the Declaration of Helsinki. Participants received a financial compensation fee after completion of each experimental day.

### Schedule of assessments and tasks

Each subject participated in three assessments performed on 3 different days, approximately at the same time of the day in order to control for the effect of circadian rhythms on muscle strength and endurance measures. The second session was conducted one to two days after the first and the third approximately 6 weeks later. A 6-week interval was chosen because it is considered the minimum duration of therapeutic exercise intervention to demonstrate muscle structural changes in reaction to muscle training [26].

In each assessment, the basic steps were as follows: 1) Basic anthropometric measurements and questionnaires that assessed subjects’ motivation and physical activity level, 2) warm-up and maximum isometric back extension tests, 3) 20 min rest, in which the surface EMG electrodes were positioned, and 4) one sustained isometric back extension test at 80% of maximum for 30 s. All tests were supervised by 3 experienced examiners (CS, MW, RH). Assessments of the psychological variables and physical activity levels were supervised by a certified clinical psychologist (BP). Subjects were asked to maintain their physical activity level during the six weeks of the study.

#### Instrumentation (equipment and tests)

##### Back extension dynamometer

Isometric maximum back extension torque moment was collected using a back extension device (F110 extension; DAVID® health solutions, Helsinki, Finland). This device consists of a “hip fixation mechanism” that is comprised of 5 components: footplates adjustable to lower leg length, knee pads adjustable to thigh length, a pelvic belt, a seat adjustable for height, and a dorsal back pad. For the maximum back extension test, participants were seated on the isometric machines with the longitudinal axis of their knees parallel to the seat, their trunk flexed forward at 30°, and their arms hanging relaxed to each side of their trunk. Seat height was variable and all positioning variables were standardized in accordance with the manufacturer’s recommendations. Strength gauges implemented into the test devices measured the trunk extension torque in Nm and were displayed in real time on the EVE monitor, attached to the device.

##### Sustained trunk extension test device

In order to obtain undisturbed EMG recording from the back muscles, participants performed the 30 s sustained back extension test on the “Total Trunk” (TechnoGym®, Italy) device. This back extension device consists of a “hip fixation mechanism” which is similar to that of the DAVID® device but includes a dorsal sacral pad instead of a back pad. Aside from this, it similarly had footplates adjustable to lower leg length, knee pads adjustable to thigh length, a pelvic belt, and a seat adjustable for height.

##### Surface EMG

Electromyographic signals were recorded with active double parallel-bar electrode sensors that also integrated triaxial accelerometric sensors (Model Trigno, DelSys®, Boston, MA, USA). After the skin at the electrode sites had been abraded with alcohol and, if necessary, shaved, the electrodes were positioned bilaterally over the multifidus muscle at L5, the longisimus at L2, and the iliocostalis lumborum muscle at L1, considering muscle fiber direction and the positioning recommended by the SENIAM project [27], by previous studies [25,28] and also the sensor location reported by Singh [20]. Testers were well trained in administering the sensors. Landmark locations rather than the multifidus muscle itself served to assign validity of the EMG signal. This was done because it is difficult to capture the multifidus muscle with surface electrodes. A reference electrode is not necessary with the Trigno EMG system. The sEMG signals were acquired using 6 active electrodes (DelSys®, Inc., Boston, MA, USA) that provided a total effective gain of 909 V/V ±5%, a bandwidth of 20-450 Hz and a baseline noise < 0.75 μV (RMS). The SEMG signals were sampled at 2000 Hz using a 16-bit AD/board and EMG works acquisition software (DelSys®, Inc., Boston, MA, USA). All sensors were secured to the skin by a double-sided adhesive interface.

##### Questionnaires

Ratings of subjects’ participants’ anticipatory positive and negative emotions were derived from the Avoidance Endurance Questionnaire [29] and their motivation was assessed according to guidelines in previously published work [30]. Both the rational and the procedures were described previously [31]. In brief, participants had to imagine the testing situations and rate their respective expectations on Borg Category Ratio scales, shortly before these tests were performed. Each rating on the respective Borg scale ranged from 0 (nothing at all) to 10 (extremely strong). Participants further completed the International Physical Activity Questionnaire (IPAQ) to evaluate whether they maintained their activity level over the 6 weeks between evaluations 1 and 3 [32].

### Test procedures

All data were collected between June 2011 and March 2012.

#### Maximum back extension test

After the test leader securely positioned the testee in the device and all restraining mechanisms and lever arm attachments were adjusted to the subject’s body dimensions, following the manufacturer’s recommendations, participants performed a warm-up at very low loads to familiarize themselves with the equipment and test procedures. Thereafter, they performed 2 consecutive maximum isometric contractions under supervision of the tester. Intervals between maximum test repetitions were a minimum of 15 s. If the 2 tests varied by more than 10%, or if the peak moment was achieved later than 3 s after the onset of the contraction, further trials were permitted until a consistent maximum was achieved. The best value obtained was recorded and stored. Verbal instructions and encouragement were standardized.

#### Sustained back extension test

After the electrodes had been attached to the muscles of interest and checked for function, volunteers were seated on the total trunk device using the same positioning variables that were used for the DAVID® device. After the subject had been secured in the device and all restraining mechanisms and lever arm attachments had been adjusted, the lever arm was loaded with 80% of the maximum load. With support of the tester, the participant moved her/his trunk into a 30° anteflexed trunk position. From this position the participant was encouraged to maintain the position constant for at least 30 s. The 80% maximum voluntary contraction (MVC) load in kg was calculated from the best maximum trunk extension moment (Nm) obtained from the DAVID® device. This was obtained by the mathematical product of the moment as recorded by the load cell of the dynamometer and the moment arm defined by the distance of the back restraint and the load cell.

### Signal processing

**Figure 1:**
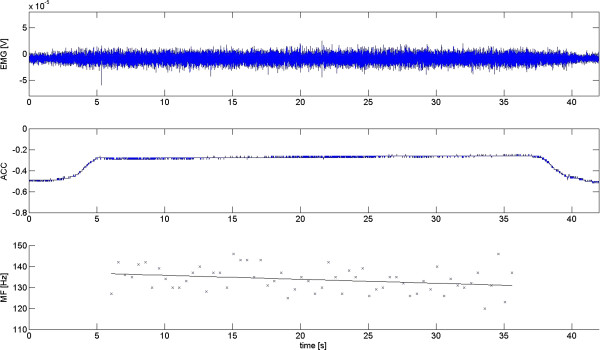
**This figure provides an example of the raw signal quality of both the surface electromyogram (upper box) and the accelerogram (mid box) recorded from the L5 left electrode site during an 80% MVC sustained back extension.** In the lower box, results of the MF-EMG processing including the MF EMG values calculated for each individual epoch and those of the linear regression analysis are provided.

After screening for and removing artefacts from the EMG signals corresponding to the 30 s sustained contraction, data were filtered between 20 Hz and 500 Hz, and Fourier transformed using a Blackman window, epoch 500 ms, 50% overlap resulting in 27 s of data at 2 Hz (i.e., 54 samples for each data set). To allow participants to stabilize the contraction/position level, the initial 3 s were omitted. A linear regression analysis was performed on MF-EMG data for each electrode site separately between 3 s and 30 s of the contraction in order to calculate the rate of decline in MF over time. The slope of the linear regression line was measured in Hz/s or, if normalised to the initial MF (intercept of the regression analysis), in %/s. An example of the signal quality and results from MF-EMG processing is provided in Figure 1.

#### Ratios and imbalance parameters

Following the previous suggestions [33] we further calculated one MF ratio for each pair of EMG electrodes at the three lumbar levels (L1, L2, and L5), adding up to three MF ratios. This was done in order to reduce the complexity of the data and because of the symmetric nature of the static fatigue task. These variables were calculated separately for each lumbar level from the sample-by-sample ratio (right-side value divided by left-side value) of the two signals of interest between 3 s and 30 s of the contraction (providing 54 ratios from 27 s of data sampled at 2 Hz). Each of the ratio values was further transformed to provide a time series corrected ratios (R) with symmetrical properties centred around 0. An average of all the transformed ratios was used to represent the segmental imbalance behaviour between the two EMG signals. The derived ratio was multiplied by 100 to represent percent difference between the right and left sides. From these local segmental ratio parameters, 2 global EMG parameters, the “uncompensated” and the “compensated” imbalance parameters, were then calculated. The “uncompensated” imbalance was defined as the mean across the three lumbar levels of the absolute value of the segmental ratios, and the “compensated” imbalance as mean of the segmental ratios across all lumbar levels [33]. The uncompensated imbalance parameter thus provides a measure of the total muscular imbalances regardless of direction (right or left), whereas the compensated imbalance parameter takes into consideration the direction of the local segmental imbalances, with a positive value indicating that right > left and a negative value indicating that left > right [33].

### Statistical analysis

#### Definition of variables

The following dependent variables were used in the analysis: MVC as an indicator of back extensor strength; individual mean bilateral IMF values as derived from the onsets of the linear regressions; the bilateral mean MF-EMG slopes expressed as absolute values and normalized to the IMF based on the 3 muscle sites to indicate the neuromuscular fatigue rate (L1 - iliocostalis lumborum, L2 - longissimus, and L5 - multifidus); the uncompensated and compensated values of individual MF-EMG muscular imbalances. The independent variables were subjects’ age (young and old) and gender. All statistical analyses were done using the software package R® [34].

#### Differences between age-specific groups

Histogram plots and Wilk Shapiro tests verified that data were normally distributed. Descriptive statistics served to summarize the participants’ characteristics. To evaluate whether maximum back extension moment or EMG fatigue variables differed between the two age-specific (and gender specific) groups, two way ANOVAs (two age and gender groups), for both the first test day and for the average values of all 3 test days, were carried out on each outcome variable. In addition, we bootstrapped the mean differences for age and gender.

#### Reliability of EMG fatigue measurements

As classical reliability analysis-based intraclass correlation coefficients (ICC) [35] would not distinguish between different sources of measurement errors, Generalizability Theory (G-Theory) [36-38] served to examine reliability of the EMG measures. Using a “multi-factorial random-effects ANOVA” model that included several sources of measurement error related to “subject, day, side, subject x day, and subject x side”, the absolute SEM values as well as the respective coefficients of dependability (D), which is a type of ICC with the corresponding absolute error variances and, consequently, the absolute standard error of measurements were calculated. Noteworthy, G-theory distinguishes between absolute and relative decisions: absolute decisions are criterion-referenced and occur if the subject's measurement results are independent of the performance of other subjects. ICC [2,1] as calculated by Shrout and Fleiss [35] would only consider relative, norm-referenced decisions, thereby focusing on the relative ordering of the subjects.

## Results

Out of 86 subjects included in the study, a total of 83 participants completed all tests. Two of the subjects were disabled in motor vehicle accidents and one subject reported severe back pain on the second test day and thus refused re-evaluation (on test day 2 and 3). Measurements were repeated after 1.9 (±2) and 43.9 (±16.4) days. The mean maximum back extension torque was significantly lower in females than in males. Older persons demonstrated an overall lower maximum back extension torque than younger ones, but age-specific differences did not demonstrate significance (Table 1).

**Table 1:** Descriptive data of participants

**Average from 3 test days**		**Mean (SE)**	**Age**	**Gender**	**Age X Gender**	**Age and gender dependant differences**	
			**F; p-value**	**F; p-value**	**F; p-value**	**Mean (95% CI)**	
						**Age**	**Gender**
**Age (years)**	<50	33.14 (1.66)					
	>50	67.11 (1.55)					
**BMI (kg/m**^**2**^**)**	<50	24.11 (0.47)					
	>50	25.15 (0.45)					
**IPAQ**	<50	337.15 (60.25)					
	>50	424.09 (59.07)					
Position of lever arm* (degrees)	<50	47.2 (13.7)					
	>50	46.7 (10.0)					
**Lumbar/Thoracic (Nm):**							
**Extension torque**	<50	242.67 (11.65)	02.00; 0.16	74.81; < 0.01	0.04; 0.84	23.98 (-5.66; 54.71)	102.85 (80.67;125.56)
	>50	218.68 (11.19)					
**Participants’ anticipatory feelings and emotions:**							
**Positive emotions**	<50	05.23 (0.29)	00.61; 0.44	02.57; 0.11	0.01; 0.91	0.37 (-0.43; 1.19)	0.68 (-0.11; 1.48)
	>50	04.87 (0.29)					
**Negative emotions**	<50	00.63 (0.12)	01.44; 0.23	01.00; 0.32	0.61; 0.44	0.21 (-0.12; 0.54)	0.18 (-0.14; 0.49)
	>50	00.42 (0.12)					
**Motivation**	<50	05.90 (0.31)	00.30; 0.58	01.99; 0.16	0.78; 0.38	0.28 (-0.47; 1.04)	0.62 (-0.13; 1.45)
	>50	05.62 (0.27)					

*Lever arm position data at the beginning of the sustained trunk extension were acquired from an accelerometer (Trigno, DelSys, MA, Boston) attached to the lever arm in a standardized way. Note that the degrees provided represent absolute values and relate to a 30° trunk anteflexion in the dynamometer.

Means and standard error are provided. This table further provides results of two-way ANOVAS (age, gender) of data from psychological testing, and the lever arm positions during the sustained contraction.

The median of the total physical activities as rated with the IPAQ at baseline was similar between age groups and these ratings remained unchanged over the 3 evaluations. Participants’ spirit and motivation to perform the test were moderate, and neither differed significantly between age groups nor did these change from test to retest after two days or after six weeks, respectively. The characteristics of the subjects for the complete sample and each of the 4 age and gender specific subgroups are presented in Table 1.

### Age and gender specific differences of MF-EMG of fatigue

Age and gender-specific intergroup comparisons of the IMF-EMG values and their respective fatigue changes, as well as the fatigue-based imbalance scores averaged from all 3 test days are shown in Table 2. IMF-EMG values were overall higher in the medially located back extensors with significant age-specific, but no gender-specific differences at L5. Isometric lumbar muscle fatigue during the 80% maximum sustained back extension was accompanied by significantly steeper absolute and normalized MF-EMG slope declines in younger than older persons. Such age-specific differences, however, demonstrated significance only at the L5 electrode recording site, or if the most negative slope or the slopes of all electrodes were considered. The fatigue related MF-EMG changes recorded from L2 revealed a tendency in favor of significantly larger changes in the younger than in the older age group. No age-specific differences were observed for the uncompensated or compensated imbalance scores. Neither the IMF-EMG nor the MF-EMG fatigue slopes revealed any significant gender-specific intergroup differences.

**Table 2:** Summary of the MF-EMG variables recorded and averaged from all 3 days

**Initial median frequency**						
**Hz average from 3 test days**		**Mean (SE)**	**Age**	**Gender**	**Age X Gender**	**Age and gender dependant differences**
		**Hz**	**F; p-value**	**F; p- value**	**F; p- value**	**Age gender mean (95% CI)**
**All electrodes**	<50	91.74 (1.75)	00.84; 0.36	00.56; 0.46	0.07; 0.79	−2.20 (-06.96; 2.48) 1.88 (-2.69; 6.37)
	>50	93.94 (1.66)				
**L5**	<50	102.81 (1.72)	07.53; < 0.01	00.29; 0.59	0.10; 0.75	−7.64 (-13.17;-2.08) -1.42 (-7.20; 4.07)
	>50	110.45 (2.23)				
**L2**	<50	90.10 (2.12)	01.33; 0.25	00.51; 0.48	0.02; 0.89	−3.28 (-08.60; 1.91) 2.06 (-3.27; 7.35)
	>50	93.37 (1.80)				
**L1**	<50	80.85 (1.77)	00.08; 0.78	00.01; 0.90	0.36; 0.55	0.58 (-03.90; 4.88) 0.14 (-4.43; 4.63)
	>50	80.26 (1.55)				
**Most negative electrode**	<50	75.25 (1.53)	00.08; 0.77	00.20; 0.66	0.01; 0.94	−0.62 (-04.65; 3.51) 0.98 (-3.19; 4.95)
	>50	75.87 (1.36)				
**Median frequency slope decline**						
**Hz/s average from 3 test days**		**Mean (SE)**	**Age**	**Gender**	**Age X Gender**	**Age and gender dependant differences**
		**Hz/s**	**F; p-value**	**F; p- value**	**F; p- value**	**Age gender mean (95% CI)**
**All electrodes**	<50	−0.20 (0.03)	05.67; 0.02	02.06; 0.16	0.41; 0.53	−0.10 (-0.17; -0.02) -0.06 (-0.14; 0.02)
	>50	−0.11 (0.02)				
**L5**	<50	−0.24 (0.04)	04.26; 0.04	00.17; 0.68	0.01; 0.98	−0.10 (-0.19; -0.01) -0.02 (-0.12; 0.08)
	>50	−0.14 (0.03)				
**L2**	<50	−0.19 (0.03)	02.79; 0.10	01.91; 0.17	0.06; 0.80	−0.08 (-0.16; 0.02) -0.06 (-0.15; 0.03)
	>50	−0.12 (0.03)				
**L1**	<50	−0.14 (0.03)	03.79; 0.06	00.55; 0.46	0.62; 0.43	−0.07 (-0.14; -0.01) -0.03 (-0.10; 0.04)
	>50	−0.07 (0.02)				
**Most negative electrode**	<50	−0.46 (0.04)	08.78; < 0.01	00.57; 0.45	0.22; 0.64	−0.15 (-0.24; -0.06) -0.04 (-0.15; 0.06)
	>50	−0.31 (0.03)				
**Median frequency slope decline normalized to initial median frequency**						
**%/s average from 3 test days**		**Mean (SE)**	**Age**	**Gender**	**Age X Gender**	**Age and gender dependant differences**
		**%**	**F; p-value**	**F; p-value**	**F; p- value**	**Age gender mean (95% CI)**
**All electrodes**	<50	−0.21 (0.03)	05.14; 0.03	01.47; 0.23	0.19; 0.66	−0.10 (-0.18; -0.02) -0.06 (-0.14; 0.03)
	>50	−0.12 (0.02)				
**L5**	<50	−0.26 (0.04)	06.40; 0.01	00.17; 0.68	0.01; 0.98	−0.13 (-0.23; -0.04) -0.02 (-0.13; 0.08)
	>50	−0.12 (0.03)				
**L2**	<50	−0.20 (0.03)	02.35; 0.13	01.62; 0.20	0.16; 0.69	−0.07 (-0.16; 0.03) -0.06 (-0.15; 0.04)
	>50	−0.13 (0.03)				
**L1**	<50	−0.16 (0.03)	03.13; 0.08	00.52; 0.47	0.75; 0.39	−0.08 (-0.16; 0.00) -0.03 (-0.11; 0.05)
	>50	−0.08 (0.02)				
**Most negative electrode**	<50	−0.46 (0.03)	09.15; < 0.01	00.19; 0.66	0.01; 0.99	−0.15 (-0.23; -0.05) -0.02 (-0.13; 0.07)
	>50	−0.32 (0.03)				
**Median frequency fatigue right - left imbalances**						
**Hz average from 3 test days**		**Mean (SE)**	**Age**	**Gender**	**Age X Gender**	**Age and gender dependant differences**
		**%**	**F; p-value**	**F; p- value**	**F; p- value**	**Age gender mean (95% CI)**
**Uncompensated**	<50	8.21 (0.42)	00.01; 0.92	00.10; 0.75	2.35; 0.13	0.00 (-1.43; 1.22) 0.14 (-1.18; 1.48)
	>50	8.22 (0.53)				
**Compensated**	<50	−1.63 (0.79)	00.16; 0.69	06.64; 0.01	0.62; 0.43	−0.49 (-2.81; 1.97) 2.96 (0.65; 5.13)
	>50	−1.14 (0.95)				

This table further presents the results of the 2 way ANOVAS (age, gender) as well as those of the subsequent intergroup comparisons.

Results from the statistical analysis that considered data from the first test day yielded similar results (data not shown).

### Retest reliability of MF-EMG fatigue in older relative to young persons

Both absolute reliability of the initial MF-EMG as assessed by the D-coefficient and the absolute SEM as calculated for the different bilateral recording sites separately were overall small and similar between young and older persons. Moreover, absolute SEM values were unanimous between the different recording sites, or if the electrode with the most negative fatigue slope was considered.

Absolute SEM values of both the non-normalized and the normalised MF-EMG fatigue slopes were overall high and varied between 28% for the most negative electrode and 100% at L1 in elderly participants, if these values were considered in terms of their respective fatigue slope declines. Although these SEM values were smaller in elders, their relative values related to the respective fatigue slope declines were higher than in younger persons, except for the recording with the most negative slope.

Since the D-coefficient is a type of ICC we can apply the common conventions: ICCs in the range of .4 to .6 are moderate, those in the range of .6 and .7 are good, and those above .75 are excellent. On the basis of these criteria, relative retest reliability was found to be good to excellent for all the variables and recording sites tested. There was no major difference in the ICCs between the different recording sites in both age groups.

ICC values of both the absolute and the normalized MF-SEMG fatigue slopes indicated good to excellent relative reliability for all back muscles tested. ICCs of the L5 recordings sites were found to be lower, but still indicated good reliability in the older group. However, ICCs of the recording site that displayed the most negative EMG slope were higher in elders. All the ICC and SEM values calculated for the different EMG recording sites are provided in Table 3.

**Table 3:** Summary of the reliability analyses of the G-theoretic approach

		**D-value**	**SEM**	**Error variance**
			**absolute**	**absolute**
**Initial MF**				
**All electrodes**	<50	0.70	5.98	35.80
	>50	0.59	7.01	49.11
**L5**	<50	0.83	4.23	17.93
	>50	0.87	4.28	18.33
**L2**	<50	0.87	4.85	23.53
	>50	0.71	5.22	27.22
**L1**	<50	0.84	4.21	17.70
	>50	0.84	3.31	10.94
**Most negative electrode**	<50	0.84	4.30	18.52
	>50	0.82	3.73	13.92
**MF slope declines**				
**All electrodes**	<50	0.70	0.11	0.012
	>50	0.69	0.08	0.006
**L5**	<50	0.74	0.10	0.011
	>50	0.74	0.08	0.006
**L2**	<50	0.79	0.09	0.008
	>50	0.81	0.08	0.006
**L1**	<50	0.75	0.08	0.007
	>50	0.62	0.06	0.004
**Most negative electrode**	<50	0.64	0.12	0.015
	>50	0.71	0.09	0.008
**MF slope declines normalized to initial MF**				
**All electrodes**	<50	0.69	0.12	0.014
	>50	0.74	0.08	0.006
**L5**	<50	0.79	0.11	0.012
	>50	0.74	0.08	0.006
**L2**	<50	0.79	0.09	0.009
	>50	0.84	0.08	0.006
**L1**	<50	0.72	0.10	0.010
	>50	0.62	0.08	0.006
**Most negative electrode**	<50	0.60	0.13	0.016
	>50	0.78	0.09	0.008

D-values are a type of an Intraclass Correlation coefficient (ICC), absolute SEM = absolute square root of the error variance.

Note that for the individual MF-EMG measures, the type of decision is absolute.

## Discussion

Estimating both muscle fiber composition and neuromuscular function with the MF-SEMG fatigue method may, if sensitive to age and of sufficient reproducibility, be a valuable tool for identifying persons at risk for sarcopenia. Furthermore, this measure could contribute important information that would aid in the implementation, goal setting and outcome monitoring of rehabilitative and preventive programs in elderly patients. As such, results of this research revealed that 1) initial MF-EMG values of the median back muscles were higher and the MF-EMG fatigue slopes were less pronounced in persons older than 50 years of age, if the L5 recording site or the most negative MF-EMG slope, or if all the slopes of lumbar electrodes, were considered, 2) absolute retest reliability was low, whereas relative reliability demonstrated overall good to excellent reproducibility for this EMG measure in the older group, and 3) that reliability values observed in the older group were comparable to those observed in the younger one.

Assuming a sarcopenia-induced higher percentage of fatigue-resistant muscle fibers that would dispose of slower muscle fiber conduction velocities than fast-fatiguing ones [10,11], we hypothesized the initial MF-EMG values of the back extensors to be lower in older subjects. Lower frequencies were especially anticipated for recordings with significantly smaller MF-EMG fatigue declines in the older participants in the case that back extension torques and tissue layers between electrodes and muscles were comparable between the two age groups. However, the initial MF-EMG values of the medial back extensors observed in this study were higher in elders, despite their respective MF-EMG fatigue changes being clearly less pronounced, their body mass indexes not significantly higher, and back extension torques well comparable between age groups. This exciting finding may be explained by two mechanisms. The first of these mechanisms refers to degenerative changes in the spine that likely occur physiologically, with advancing age, and the related changes in mechanical lengthening states of the short, medially located mono- and oligospinal back extensors. As compared with lengthened muscles, shortened muscles have a higher EMG signal per unit force and exhibit a shift in the power spectrum toward higher frequencies [39]. Thus, degenerative spine alterations with a narrowing of the intervertebral spaces may have led to comparably shorter lengthening states of the paravertebral back extensor muscles in the older participants, thereby inducing higher than expected IMF-EMG values for the medial back muscles. The second and more likely proposed mechanism would consider a relatively high training status of the fatigue-resistant muscles fibers in the medial back extensors of the older persons tested in this study. Training performed by elderly preferentially induces, unlike in young persons, type I fiber muscle hypertrophy and increases in muscle fiber contraction properties [40,41], which could relate to increased muscle fiber conduction velocities of these fibers. Indeed, 6 weeks’ of training improved muscle fiber conduction velocities in low threshold motor units of the quadriceps muscle [42]. These low threshold motor units may reasonably be assumed to be predominantly comprised of fatigue-resistant muscle fibers. Consequently, a relatively high back extensors’ training status in the elderly of this study may have likely caused higher than expected IMF values in the L5 and L2 recordings. This mechanism would also fit the concept that surviving muscle fibers in even activity limited elderly persons were able to partially compensate for muscle strength deficits at the whole muscle level in order to maintain optimum force-generating capability [2,43]. This concept would further support the clinical relevance of the IMF-EMG rather than EMG slope declines, if spectral EMG was used as an outcome monitoring tool in elderly.

Unlike previous work [13,20], findings of this study successfully demonstrated that the MF-SEMG muscle fatigue slopes were of sufficient discriminant sensitivity to distinguish between normal and sarcopenic back muscle structure and function if a test protocol had been administered that was specific to back extensors [21] and best controlled for neuromuscular activation and muscle metabolic confounders of the spectral EMG [24]. Using this paradigm, the MF-EMG fatigue slope declines observed from the multifidus muscles (L5) or from the recording with the most negative slope or from the recordings of all electrodes clearly distinguished between young and sarcopenic muscle function. These findings seem to confirm those from a previous study that compared both the EMG fatigue changes and the absolute endurance times of back extensors recorded at 3 different submaximum loads (30%, 50% and 70% MVC) between young (mean age 21 years) and older persons (mean age 61 years) [13]. Unlike the low and mid-loaded task, the high-loaded task was accompanied by significantly less pronounced MF-EMG fatigue in both the multifidus and the longissimus muscles in the older group, although absolute endurance times had been comparable between the two age groups. Our findings of age sensitive MF-EMG fatigue slope declines in back extensors, however, appear to contrast somewhat with those of another previous study [20]. The Sing study failed to demonstrate age-specific MF-EMG fatigue differences for the longissimus muscle that had been recorded at the L3 level during a 60% MVC sustained back extension in an erect standing posture. As the present study found, the longissimus muscle was less sensitive to age-specific effects than the multifidus. These findings would in part fit the negative ones of the Singh study. Factors like age-dependent alterations of load sharing between the medial and lateral back extensors or the upper and lower medial back extensors [44], alteration of the sagittal alignment of the lumbar spine with advancing age despite the standardized back positioning [45], and/or alterations in trunk stiffness due to increased co-contraction of the medial and lateral trunk muscles as well as the abdominals in elderly, may have contributed to the attenuation of age-specific MF-EMG fatigue effects in this muscle.

### Reliability

In this study the G-theory was used for assessing reliability of measurements. It estimates several sources of error and acknowledges and allows for variability in the EMG assessment conditions that may reasonably be expected to occur in everyday clinical practice. In our setting, we had multiple sources of measurement errors: subject, day, side, subject x day, and subject x side. In addition to the specification of such multiple variance components, G-theory would further take into account how the consistency of measurements might change if the EMG measures were used to make absolute versus relative decisions: absolute decisions are criterion-referenced and occur if the subject's measurement results are independent of the performance of other subjects; relative decisions are norm-referenced and focus on the relative ordering of the subjects [36-38]. Classical reliability analysis based on intraclass correlation coefficients (ICC) [35] would not distinguish among different sources of measurement errors. In fact, previous studies that had tested reproducibility of MF-EMG slopes of back muscles with classical statistical approaches found ICCs and SEMs that suggested this classical method of little clinical use [23,45,46].

Administering the G theoretic approach to the data of this study, findings revealed that the test retest reliability of the IMF-EMG values was excellent for the different back muscles in both age groups, suggesting this measure of sufficient sensitivity to changes. This is supported by the findings of several reliability studies performed in young healthy persons that demonstrated the initial median frequencies of sustained contractions as highly stable and only minimally affected by load [24,25,28,45]. As sagittal positioning of the spine may affect initial MF-EMG in a significant way [45], special care has to be taken in controlling hip and spine posture in back extensions as changes in the lumbar sagittal profile may significantly alter the MF-EMG values, and thus impair the stability of this measure from test to test. Seated position and the 30° trunk flexion as used in our test protocol seemed highly appropriate to allow reliable MF-EMG measurements [31].

Although this study found good to excellent relative reliability for the MF-EMG slope declines with no relevant differences between the two age groups, SEM values, if related to the slope declines, indicated this measure as highly variable in both age groups. Even the most stable recording, which was derived from the most negative slope varied by 28% in both age groups, indicative of limited clinical value. These observations concur with those from previous studies that found the back extensor spectral fatigue slope declines taken from the recording with the steepest negative slope most stable [25]. This attests to the belief that the MF-EMG fatigue slope declines are of little clinical value if used as an outcome measure in clinical every day practice [23,25].

### Limitations

This study included healthy elderly persons that were eager to participate. Frail elderly individuals or those older subjects who would be less motivated to do the tests were not included. When compared to young persons, we would expect to observe clearly lower maximum back extension torques in older persons. This may not only be related to muscle weakness but also to lower motivation to put forth their maximum effort. As MF-EMG fatigue changes depend on the percentage of the MVC used for a sustained contraction [33], findings of this study may not be representative of all older persons. Standard motivation protocols developed with a psychologist may help in part to overcome these limitations as had been demonstrated in a recent study [31].

In each of the 3 examination days, researchers assessed maximum back extension torque. These back extension torques varied by approximately 10% between days in young as well as in older persons when they had been tested with a David F110 device [David Health solutions, Helsinki, Finland] [31]. Thus, the variability of the 80% load used for the sustained back extension might have contributed to the lower stability of the MF-EMG slopes as their declines were dependent on the relative loading of a muscle. Administering the same load for reliability testing might have improved the SEM values of the EMG-slopes. However, such a procedure would be without clinical relevance, as outcome monitoring after the termination of a preventive or rehabilitation program usually also required reassessment of maximum strength performance.

It remains uncertain whether or not the age-specific differences in the L5 recordings or at the site with the least negative slope would exclusively reflect age-specific physiological adaptations in muscle fiber composition, or if other mechanisms may have contributed to these findings. One likely mechanism considers the increased synergistic contribution of the hip flexors to the back extension torque in elderly persons, which thereby reduces the load of the multifidus and iliospinalis muscles. In fact, both the psoas major and the quadratus lumborum were shown to contribute to back extension and were activated to a higher extent in lumbar lordosis than kyphosis [47,48]. However, due to the greater distance of the hip flexors than the back extensors to the electrode recording sites, their activity may be assumed either not represented or only weakly represented in the SEMG. Moreover, the lordotic posture of the lumbar spine in a seated and trunk anteflexed position would be expected to be similar between age groups, or even less pronounced in the elderly. Thus, a redistribution of lower loading of the back extensor muscles has unlikely facilitated the flattening of the MF-EMG fatigue slope declines in the L5 recordings of our elderly participants.

## Conclusions

The MF-SEMG fatigue method is sensitive enough to demonstrate functional alterations of aging back muscles. As reliability of the initial MF-EMG was found clinically acceptable and relative reliability of the MF-EMG fatigue declines to be good to excellent, this method may not only be deemed as a simple and easily available diagnostic screening tool for persons at risk for sarcopenia, but also as an outcome monitoring tool in elders. Future research will need to clarify how this objective neuromuscular assessment technique may relate to traditional measures of muscle performance and structure, and whether or not abnormal findings assessed with this technique would also reliably predict an increased risk of disablement in these persons.

## Competing interests

The authors declare that they have no competing interests.

## Authors’ contributions

TK concepted and designed the study, supervised data collection, and drafted the manuscript. RH participated in the design of the study, data collection, performed all statistical analyses, and participated in drafting the manuscript. CS participated in the design of the study, data collection, statistical analyses, and participated in drafting the manuscript. SR participated in data processing, statistical analyses and in drafting of the manuscript. PM participated in the design of the study, supervised the statistical analysis and data interpretation, and participated in drafting the manuscript. JK concepted and designed the study, supervised data acquisition and processing, participated in data analysis and interpretation, and participated in drafting the manuscript. BP participated in the conception and design of the study, supervised psychological data collection and processing, and participated in drafting the manuscript. GE concepted and designed the study, participated in the supervision of data collection and processing, participated in data analysis and interpretation, drafted and finalized the manuscript. All authors read and approved the final manuscript.
